# Radiation testing of the AeroForm co_2_-based breast tissue expander implant

**DOI:** 10.1186/1748-717X-8-235

**Published:** 2013-10-11

**Authors:** James L Rembert, Roxana Heitz, Adam Hoffman

**Affiliations:** 1 Alta Bates Summit Comprehensive Cancer Center, 2001 Dwight Way, Berkeley, CA 94704, USA; 2 AirXpanders, Inc, 1047 Elwell Court, Palo Alto, CA 94303, USA

**Keywords:** Tissue expander, Breast reconstruction, Radiation therapy, Ionizing radiation, Electronics, Circuit

## Abstract

**Background:**

Tissue expanders are used in breast reconstruction after mastectomy to stretch the remaining tissue to create space for placement of permanent breast implants. The AeroForm™ Tissue Expander, developed by AirXpanders™ Inc., contains electronic components designed to activate the release of carbon dioxide from an internal reservoir to inflate the expander. Breast cancer patients who undergo mastectomy and tissue expander/implant-based breast reconstruction may require radiation therapy at doses up to 50–60 Gy while the expander is in place. The ionizing radiation used in postmastectomy radiation therapy interacts with electronic components in medical implants, which may cause degradation in performance above certain levels. Most commercial electronic components used in medical devices, such as complementary metal-oxide semiconductor or bipolar integrated circuits can withstand radiation levels in the 50 Gy range without any performance degradation. Beyond this level, the performance may still be sufficient to guarantee functionality, but this needs to be confirmed at the system and electronic circuit level. We assessed the impact of radiation levels up to 75 Gy on 32 AeroForm™ Tissue Expanders (AirXpanders, Inc., Palo Alto, CA USA) and on the associated internal printed circuit assemblies.

**Findings:**

The electronics inside the AeroForm™ Tissue Expander implant continued to function properly after exposure to radiation levels up to 75 Gy, which is well above the maximum total dose level typically used in postmastectomy radiation therapy.

**Conclusions:**

Standard postmastectomy radiation therapy doses do not damage or affect the functionality of the AeroForm™ Tissue Expander.

## Findings

### Background

Following mastectomy, immediate breast reconstruction is the standard of care in many clinical settings because of the associated psychological benefits to the patients [1-3]. Typically, patients who select implant-based breast reconstruction require expansion of the remaining tissue with saline expanders, which involves many disruptive and often painful saline injections over several months at the surgeon’s office. AirXpanders (AirXpanders, Inc., Palo Alto, California USA), in an effort to provide the patient with a more comfortable, gradual tissue expansion process that they control, has manufactured and is investigating a breast tissue expansion system consisting of an implantable tissue expander (AeroForm™) and a handheld radio-frequency dosage controller. The dosage controller communicates with the expander and allows the patient to administer 10 cc doses of CO_2_ from a reservoir within the expander; pre-clinical and feasibility studies have been reported [4,5].

Approximately 55% of women with high-risk breast cancer undergo radiation therapy postmastectomy [6]. Radiation protocols vary but in cases of immediate reconstruction, radiation may be administered during the reconstruction process with the tissue expander in place. In light of the fact that AeroForm expansion depends on the proper functioning of the implant electronics, we chose to test the electronics of the expander implant and its printed circuit assemblies (PCAs) in conditions that simulated as well as exceeded the radiation exposure encountered in a clinical therapeutic setting.

## Methods

### Radiation parameters

The cumulative doses typically used in postmastectomy radiation therapy are 50–60 Gy and are administered in fractions of 1.8-2.5 Gy/day over several weeks to minimize damage to tissue [7,8]. Modern clinical radiotherapy utilizes megavoltage X-rays produced by a linear accelerator with energies typically ranging from 4–20 MV [9-11]. Prior to the production of these linear accelerators, Cobalt-60 gamma ray sources were used, which emit gamma rays with energies of 1.17 MV and 1.33 MV. X-rays with energy above 5 MV affect silicon devices in a similar manner as equivalent radiation from Cobalt-60 (Co-60) gamma rays [12].

### Electrical parameters

Threshold voltage (one of the electrical properties of metal-oxide-semiconductor field-effect transistor (MOSFET) devices) is affected by radiation exposure in a predictable pattern [13], and indeed MOSFETs have been used as dosimeters in various medical applications [14]. Threshold voltage variation is a measure of the impact of radiation on the tissue expander electronics. In the AeroForm electronics, the threshold voltage of a system-critical MOSFET (FK390601; Panasonic Corporation, Osaka, Japan) is required to be greater than 806 mV to guarantee full system performance, based on various parameters within the electronics. Analyzing this metric offers an understanding of how the system performs compared to this theoretical failure point.

### Procedure

We contracted a third-party commercial radiation testing laboratory (Aeroflex RAD, Inc., Colorado Springs, Colorado USA) to perform total ionizing dose (TID) tests of AeroForm tissue expanders and their PCAs using a Co-60 radiation source [15]. The dose rate was 6 Gy/minute (600 MU/min) to simulate the highest commonly used dose rate on commercial linear accelerators. The laboratory exposed test samples in air at different increments up to a total clinical dose of 50 Gy plus increments up to 1.5 times that dose to determine the total ionizing dose effects on the device’s function and that of its electronic components. Since little-to-no “healing” (or annealing) occurs in electronics between consecutive fractions [16], we considered only total cumulative doses in the test. Total dose was determined by the amount of time units were exposed at the specified dose rate. Scattering dose, single-event dose, and transient dose effects were not considered in this study.

The laboratory exposed four tissue expander test samples to each of the following seven TID radiation levels: 10 Gy, 20 Gy, 30 Gy, 40 Gy, 50 Gy, 60 Gy and 75 Gy. The lab also exposed an additional four tissue expanders to 50 Gy, for a total sample of 32 tissue expanders. In addition to the expanders, sets of four individual PCAs (totaling 28 PCAs) were also subjected to the same dose levels.

Prior to irradiation, we tested each tissue expander and PCA according to the manufacturer’s custom functional test to verify functionality of the products. Testing involved a comprehensive simulation of patient and physician use: (1) establish communication between the dosage controller and the tissue expander as evidenced by light and sound feedback from the dosage controller, (2) verify that sufficient coupling strength (coupling voltage greater than 1.15 V) can be achieved at a distance of 1–2.5 cm between the dosage controller and the tissue expander, and (3) verify the delivery of CO_2_ doses. Testing also measured design-specific parameters such as time constants, MOSFET threshold voltages, and other voltage and current levels. The test results were used to assess the margin in system performance given the radiation effects. Throughout the process, the electronics were handled in an electrostatic discharge (ESD)-safe environment, using appropriate ESD protection equipment.

The laboratory provided dosimetry information for each radiated set, and all parts were returned to us packed in dry ice to prevent any annealing in the components during shipping. Upon receipt of the product, we allowed the samples to return to room temperature and then repeated the manufacturer’s tests twice for each unit received: one set of tests was performed within 72 hours of irradiation (immediate); the other was performed between 26 and 30 days after irradiation (delayed). The two sets of tests were identical to the pre-irradiation tests.

## Results

Each of the 32 tissue expanders passed all three sets of functional tests (pre-irradiation, immediate and delayed) and performed according to product specifications. All expanders were able to establish communication with the dosage controller, achieve coupling voltage greater than 1.15 V (Table 1), and deliver three CO_2_ doses. There were no significant differences in coupling voltage with respect to test or radiation level (two-factor ANOVA, F_test_ = 2.455, p = 0.1278, F_level_ = 1.794, p = 0.1830).

**Table 1:** Coupling voltage before irradiation, within 72 hours of irradiation, and 26–30 days after irradiation

	**Pre-irradiation**	**Immediate**	**Delayed**
Mean	1.228	1.210	1.219
σ^2^	0.027	0.033	0.032
Min	1.18	1.16	1.17
Max	1.32	1.3	1.31

**Figure 1:**
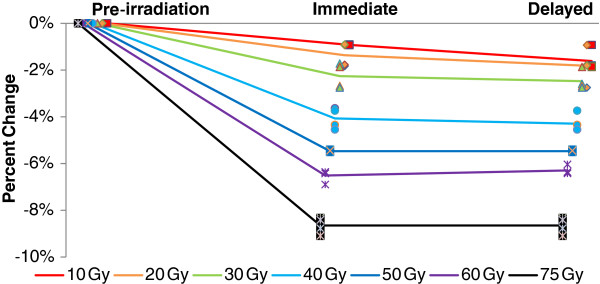
Average percent change (± standard error) in threshold voltages of a MOSFET.

**Figure 2:**
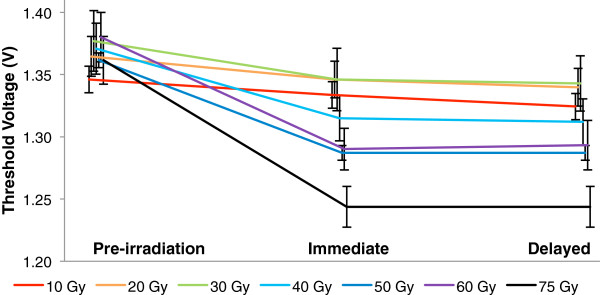
The average threshold voltage (± standard error) of MOSFET in-circuit.

Figure 1 shows the percent change in threshold voltages of a MOSFET whose function was an indicator of system performance on the PCAs, before radiation exposure, within 72 hours post-irradiation, and at 26–30 days post-irradiation. Figure 2 shows the average threshold voltage of this MOSFET in-circuit (± standard error) on an absolute scale (rather than a relative scale). There were significant effects of test (pre-irradiation, immediate, delayed), and radiation level (two-factor ANOVA, F_test_ = 16.94, p = 0.0003, F_level_ = 3.8457, p = 0.0225). Threshold voltages showed a statistically significant decrease after radiation exposure (paired *t*-test for means, t = 3.8552, p = 0.0084), but did not continue to change between the first and second post-irradiation tests (paired *t*-test for means, t = 1.6859, p = 0.1428).

A fair degree of component-to-component variation in threshold voltage was evident; however, in all cases, the threshold voltage remained well above the minimum theoretical voltage to maintain system functionality post-irradiation (806 mV). Of note, the manufacturer of this MOSFET guarantees an initial threshold voltage of 0.9 V–1.5 V under normal conditions, and the initial threshold voltage for all of these components was at the upper end of the guaranteed range. In a theoretical worst-case scenario, where the highest observed percent change (-9.091%, observed at 75 Gy) would occur in a component whose threshold voltage starts at 0.9 V, the resulting threshold would still be above the minimum allowable (818 mV vs. 806 mV). The worst-case scenario at 50 Gy (-5.505% drop) would exhibit a 5.2% margin (850 mV vs. 806 mV).

**Figure 3:**
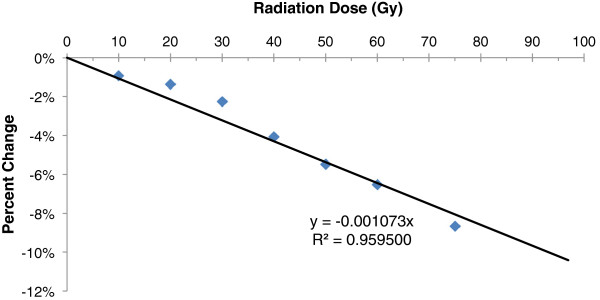
Correlation between threshold voltage change and radiation dose.

There was a strong linear correlation between threshold voltage change and radiation dose (R^2^ = 0.9595, slope = -0.1073% per Gy, assuming y-intercept at zero) (Figure 3). The theoretical maximum radiation dose under which the system would maintain functionality is 97.34 Gy, as extrapolated from the slope in the figure and assuming a worst-case initial threshold voltage of 0.9 V (percent change from 0.9 V = (0.806-0.9)/0.9 = -0.001073*97.34).

## Discussion

The AeroForm tissue expanders and associated electronics performed to their specified functionality when tested under simulated harsh radiation environments. The first test, performed within 72 hours of irradiation, provided a worst-case scenario measurement, while the second test, performed 26–30 days after irradiation, more closely represented typical clinical use wherein the patient would resume tissue expansion several weeks after undergoing radiation therapy. The described functional testing and results predicate successful operation of the AeroForm tissue expander in patients undergoing postmastectomy reconstruction with adjuvant radiation therapy.

The results of this testing are not intended as a recommendation for the use of radiation therapy in patients with an implanted tissue expander. The decision regarding radiation therapy in patients with a tissue expander in place should be made by the surgeon and radiation oncologist.

## Abbreviations

ANOVA: Analysis of variance; Co-60: Cobalt-60; ESD: Electrostatic discharge; MOSFET: Metal-oxide-semiconductor field-effect transistor; PCA: Printed circuit assembly; TID: Total ionizing dose.

## Competing interests

Dr. Rembert occasionally consults for AirXpanders, Inc., the sponsor of the study. Dr. Heitz and Dr. Hoffman are employees of AirXpanders, Inc.

## Authors’ contributions

JR was responsible for the analysis and interpretation of the data, revised the drafts for intellectual content, and approved the final version to be submitted for publication. RH was responsible for designing and coordinating the study, performing data collection, and editing drafts of the manuscript. AH was responsible for data collection and analysis, and revising the manuscript. All authors read and approved the final manuscript.

## Authors’ information

J.R. is a member of the American Board of Radiology, Radiation Oncology. R.H. has been a member of the Institute of Electrical and Electronic Engineers (IEEE) since 2002. A.H. has been a member of the Tau Beta Pi Engineering Honor Society since 2000.
